# Genome-wide identification and characterization of ALKB homolog gene family in wheat (*Triticum aestivum L.*)

**DOI:** 10.3389/fpls.2025.1544879

**Published:** 2025-03-18

**Authors:** Pengkun Wang, Tianye Zhang, Zechi Wu, Lei Yu, Pingan Liao, Jian Yang, Bingjian Sun

**Affiliations:** ^1^ The Engineering Research Center for Plant Health Protection Technology in Henan Province, College of Plant Protection, Henan Agricultural University, Zhengzhou, Henan, China; ^2^ State Key Laboratory for Managing Biotic and Chemical Threats to the Quality and Safety of Agro-products, Key Laboratory of Biotechnology in Plant Protection of Ministry of Agriculture and Rural Affairs (MARA), Key Laboratory of Green Plant Protection of Zhejiang Province, Institute of Plant Virology, Ningbo University, Ningbo, China; ^3^ Luohe Academy of Agricultural Sciences, Luohe, Henan, China

**Keywords:** AlkB homologs, wheat, m^6^A RNA methylation, demethylase, abiotic stress

## Abstract

**Introduction:**

N^6^-methyladenosine (m^6^A) is the most prevalent posttranscriptional modification in eukaryotic mRNAs. AlkB homologs (ALKBHs) are involved in plant responses to stress by modulating m^6^A methylation. However, homologous genes in wheat remain largely uncharacterized.

**Methods and results:**

In this study, 30 *ALKBH* genes were identified in wheat, and analyzed their physicochemical properties. The phylogenetic analysis allowed the classification of these genes into seven distinct subfamilies. Additionally, their conserved domains, motif compositions, gene structures, chromosomal localization, and synteny, and the predicted cis-acting elements within their promoters were examined. Expression analysis revealed that *TaALKBH9B*-5 exhibited the highest expression and its demethylase activity was investigated. Furthermore, *TaALKBH9B*-5 was significantly upregulated in response to abscisic acid treatment and cold stress, indicating a positive regulatory trend.

**Discussion:**

In conclusion, this study provides a comprehensive genomic assessment of the *TaALKBH* gene family and offers a theoretical framework for understanding the role of *TaALKBH9B* in the response to abiotic stress in wheat.

## 1 Introduction

N^6^-methyladenosine (m^6^A) represents one of the most abundant and widespread post-transcriptional RNA modifications in eukaryotes (Zhang et al., 2022). In plants, the methylation levels of m^6^A in messenger RNAs (mRNAs) are modulated by three principal components: writers, erasers, and readers. The writer complex, which includes methyltransferases such as MTA (human homolog of METTL3), MTB (METTL14), FIP37 (WTAP), VIRILIZER, HAKAI, and FIONA1, facilitates the addition of m^6^A to RNA (Yue et al., 2022b; Růžička et al., 2017; Wang et al., 2022; Yue et al., 2022a). m^6^A reader proteins, including ECT2, ECT3, ECT4, and CPSF30-L, possess a YTH domain that recognizes and binds to m^6^A-modified mRNAs, thereby regulating gene expression and impacting various biological functions (Wei et al., 2018; Arribas-Hernández et al., 2018; Hou et al., 2021). Demethylases from the AlkB homolog (ALKBH) family function as erasers, removing m^6^A from mRNAs (Martínez-Pérez et al., 2017). A substantial body of evidence suggests that RNA modifications critically regulate mRNAs, influencing plant growth, development, and stress response (Zhou et al., 2019; Amara et al., 2022). In a recent report, researchers have discovered that the natural variation in the wheat demethylase *TaMTB* (SNP176A/C) influences virus infection; however, it does not affect wheat growth and yield (Zhang et al., 2022). Furthermore, the wheat reading protein *TaECT9* exhibits a significant upregulation in response to drought stress (Pan et al., 2024). To date, there have been no documented studies concerning the members of the *ALKBH* gene family in wheat.

The ALKBH family represents the sole group of enzymes in plants recognized for their m^6^A demethylase activity. The first member of this family, ALKBH, was identified in 1983 as a DNA repair protein in *Escherichia coli* (Kataoka et al., 1983). Following this discovery, the obesity-associated protein (FTO) (Jia et al., 2011) and *ALKBH5* (Zheng et al., 2013) were identified as m^6^A demethylases, demonstrating that m^6^A methylation is reversible and influences gene expression. Since that time, numerous ALKBH family genes have been identified across various plant species, elucidating their roles in the regulation of growth and developmental processes. In *Arabidopsis thaliana*, *AtALKBH10B* has been demonstrated to demethylate m^6^A from mRNAs and to facilitate m^6^A demethylation at the FLOWERING LOCUS T (FT), SQUAMOSA PROMOTER BINDING PROTEIN-LIKE 3 (SPL3), and SPL9 during the flowering process, thereby specifically regulating the transition to flowering (Duan et al., 2017). In tomatoes, *SlALKBH2* interacts with the *SlDML2* transcript, influencing the ripening of tomato fruit (Zhou et al., 2019). Furthermore, an expanding body of research has underscored the significant role of ALKBH family members in plant responses to abiotic stress. Studies on Arabidopsis have demonstrated that *alkbh6* mutant plants exhibit elevated levels of m^6^A and enhanced tolerance to salt, drought, and heat stress. This resilience is attributed to the downregulation of abscisic acid (ABA)-related genes mediated by *AtALKBH6*-induced m^6^A demethylation (Huong et al., 2020). Compared with wild-type plants, transgenic plants that overexpress *ALKBH8B* presented a reduced overall m^6^A level and exhibited greater salt tolerance (Huong et al., 2022). The *alkbh9c* mutant presented delayed seed germination under salt, drought, and low-temperature stress and under ABA treatment. While salt stress or ABA treatment inhibits the growth of mutant seedlings, drought conditions enhance it. Under dehydration stress, the m^6^A levels of several positive effectors associated with drought stress in the *alkbh10b* mutant were found to be elevated, although their transcriptional abundance was lower in the mutant compared to transgenic lines (Han et al., 2023). During seed germination, the *ALKBH10B* mutant presented reduced osmotic pressure and salt stress tolerance, resulting in a salt-sensitive phenotype. In cotton, the silencing of *GhALKBH10* enhances antioxidant capacity and reduces cytoplasmic Na^+^ levels, thereby improving salt stress tolerance (Cui et al., 2022). Furthermore, the demethylase *GhALKBH10B* has been shown to increase drought tolerance at the seedling stage by decreasing the m^6^A level and the mRNA stability of genes associated with the ABA signaling pathway and calcium signaling (Zhang et al., 2024). In tomatoes, the *Slalkbh10b* mutant showed increased drought and salt stress tolerance, characterized by improved water retention, higher photosynthetic product, and proline levels, and reduced reactive oxygen species levels and cellular damage (Shen et al., 2023).

Wheat is one of the most essential food crops worldwide. However, throughout its growth cycle, wheat encounters various environmental challenges, including elevated temperatures, drought conditions, and high salinity, which impede normal growth and development, ultimately resulting in reduced yields. Numerous RNA modifications, particularly m^6^A methylation, are crucial for the response of plants to abiotic stresses. Despite this significance, research on m^6^A demethylases in wheat remains limited, with investigations into members of the ALKBH family being even scarcer. Therefore, there is an urgent need to identify and analyze the function of the wheat *TaALKBH* gene in stress resistance. The availability of several wheat reference genomes ( *http://www.wheatgenome.org/* ) provides a substantial platform for gene identification. In this study, 30 candidate genes belonging to the TaALKBH family were identified and categorized into seven distinct groups. Their evolutionary relationships, chromosomal locations, gene structures, and cis-regulatory elements were thoroughly analyzed. Furthermore, the tissue-specific expression patterns of the seven ALKBH subfamilies were preliminarily examined. *TaALKBH9B-5* was identified as a potential m^6^A demethylase, and its expression under abiotic stress was investigated. This study elucidates the *ALKBH* family genes associated with m^6^A demethylases in wheat and establishes a foundation for future research into their biological functions.

## 2 Materials and methods

### 2.1 Identification of *TaALKBH* gene family members

The protein sequences of Arabidopsis *ALKBH* family members (*AT1G11780*, *AT3G14140*, *AT3G14160*, *AT5G01780*, *AT2G22260*, *AT4G20350*, *AT4G02485*, *AT1G31600*, *AT4G36090*, *AT2G17970*, *AT1G48980*, *AT1G14710*, *AT2G48080*, and *AT4G02940*) were downloaded from the National Center for Biotechnology Information (NCBI) database ( *https://www.ncbi.nlm.nih.gov* *, accessed* on October 14, 2024) and used as Blastp templates to identify all ALKBH proteins in wheat, rice, and maize. Genomic data for wheat, rice, and maize were obtained from the Ensembl Plants Database ( *https://plants.ensembl.org/Triticum_aestivum/Info/Index* , accessed on October 14, 2024. 30 wheat homologs with Eval < 10^−6^ and %ID > 60 were screened, along with 9 rice homologs and 11 maize homologs meeting the same criteria (**Supplementary Table S1**). All candidate proteins were further analyzed using the Pfam database (https://pfam.xfam.org/, accessed on October 14, 2024) (Finn et al., 2014). Detailed information on the *TaALKBH*s, including the amino acid counts, chromosome positioning, and coding sequence lengths, was obtained from the Ensembl Plants Database (Bolser et al., 2015). The molecular weight, theoretical isoelectric point, and subcellular localization predictive of each TaALKBH protein were obtained using CELLO ( *http://cello.life.nctu.edu.tw/* , accessed on October 14, 2024) (Yu et al., 2004).

### 2.2 Multisequence alignment and phylogenetic construction

The phylogenetic tree of the wheat *ALKBH* gene family protein sequences was constructed using MEGA-X (Version: 10.2.6) software with the maximum likelihood method (ML) and 1000 bootstrap replicates (Kumar et al., 2018). The resultant analysis was visualized using TBtools.

The identified TaALKBH, OsALKBH, ZmALKBH and AtALKBH protein sequences were downloaded from the Ensembl Plants Database and imported into MEGA-X, and multiple sequence alignments were performed using the MUCLE analysis method, a rootless phylogenetic tree (1000 step repeats) was constructed by maximum likelihood method. A graphical representation of the generated developmental tree was beautified using the online tool ITOL [https://itol.embl.de//, (accessed October 14, 2024)].

### 2.3 Structural prediction of protein

The SWISS-MODEL (https://swissmodel.expasy.org/, accessed on 9 January 2025) was used to predict the structures of the TaALKBH proteins. A gene-building protein model was randomly selected from each subfamily for each species.

### 2.4 Conserved structural domains, gene structure, motif analysis, and cis-element analysis

The conserved structural domains of the TaALKBH protein sequences were identified using the Batch CD-Search program (Marchler-Bauer and Bryant, 2004). Conserved motifs of the TaALKBH proteins were identified using the online service platform MEME [https://meme-suite.org/meme/, (accessed 24 October 2024)] with up to 15 motifs (Zheng et al., 2022). Gene structure analysis of GFF data downloaded from Ensembl Plants was performed to identify UTRs, exons, and introns. The 2000 base pair region upstream of the ATG of each *TaALKBH* gene was analyzed in cis-element analysis. The PlantCARE online platform [https://bioinformatics.psb.ugent.be/, (adopted on 25 October 2024)] was used for prediction (Yang et al., 2023). The results of the above analyses were visualized using TBtools.

### 2.5 Chromosome localization, gene duplication, and calculation of *Ka/Ks* values

The physical location of each *TaALKBH* gene in the annotation of the wheat genome in the Ensembl Plants database was used to determine chromosomal mapping. The GFF data containing all chromosome length information of wheat provided by the Ensembl Plants database were used to extract the position information and gene density map of the *TaALKBH* gene on chromosomes. The three datasets were then uploaded to TBtools for chromosomal position analysis of *TaALKBHs*. The One-Step MCScanX-Super Fast program integrated with TBtools was used to analyze the replication pattern and collinearity between the *TaALKBHs* (Chen et al., 2020). The *Ka/Ks* ratio was calculated using TBtools software, and the scattering time (T) (Hurst, 2002)was calculated based on T=Ks/(2∗9.1∗10^−9) Mya.

### 2.6 Plant material, growing conditions

The materials used in this study, including Yangmai 158, were provided by Dr. Yang Jian. The wheat seeds were grown in an artificial greenhouse at 25°C for a 16 h light/8 h dark cycle, and when the wheat was grown to the three-leaf stage, the rhizomes and leaves of wheat were taken for RT–qPCR analysis.

### 2.7 RNA extraction and RT–qPCR assay

The total RNA of the Wheat samples was extracted using the HiPure Plant RNA Mini Kit (Magen, Guangzhou, China). First-strand cDNA was synthesized using the First-Strand cDNA Synthesis Kit (Vazyme, Nanjing, China). The RT–qPCR was carried out using ChamQ Universal SYBR Green Master Mix (Vazyme, Nanjing, China) on an Applied Biosystems Quant Studio 5 Flex system (Applied Biosystems, Foster City, CA, USA), and the relative expression levels of the assayed genes were calculated using the 2^−ΔΔCt^ method (Livak and Schmittgen, 2001) and the *T. aestivum* cell division cycle (*TaCDC*) gene (accession number: XM_020313450) was used as an internal reference gene (Zhang et al., 2019). Each treatment had three biological replicates and three technical replicates. The primer sequences used in this study are listed in **Supplementary Table S2**.

### 2.8 Tissue-specific expression

Wheat plants were divided into three tissue types: leaves, stems, and roots (Huang et al., 2024). One *TaALKBH* gene was randomly selected from each of the seven groups to analyze its expression in three different wheat tissues. Collect 3 replicates of each wheat tissue sample and store at -80°C until total RNA is extracted. Gene expression was determined by RT–qPCR. RT–qPCR reaction conditions: 95°C for 5 min, 1 cycle; This was followed by 40 cycles at 95°C 15 s, 58°C 20 s, and 72°C 30 s. It is then cycled at 72°C for 8 min and a single spot fluorescence detection is performed at 72°C. The results of tissue-specific expression analysis were displayed using GraphPad Prism 9. 0. 0.

### 2.9 Plasmid construction

The full length of *TaALKBH9B-5* was cloned from Yangmai 158 cDNA. 35S: *TaALKBH9B-5* was constructed using gateway technology. The primer pairs listed in **Supplementary Table S2** were used for the first PCR. A second PCR was performed using primers attB1 and attB2 and the amplification product from the first PCR as a template. These amplified products are introduced into pDONR207 by a BP reaction (BP Clonase Reaction), and then these fragments are further transferred to the target vector, respectively. Full-length *TaALKBH9B-5* was inserted into the pGEX4T-2 (a prokaryotic expression vector encoding GST) using the ClonExpress MultiS One-Step Cloning Kit (Vazyme, China). The primer sequences used in this study are listed in **Supplementary Table S2**.

### 2.10 Protoplast transformation and protein purification

Recombinant plasmid *TaALKBH9B-5*-GST and pGEX4T-2 were transformed into the Escherichia coli strain Rosetta-gami (DE3). The proteins were purified following the manufacturer’s instructions for Ni-NTA 6FF Sefinose (TM) Resin Kit (BBI C600332). 35S: *TaALKBH9B-5* recombinant plasmid was transfected into wheat protoplasts using a PEG-mediated transformation method (Bio-Rad, Hercules, CA, USA). The transfected mesophyll protoplasts were cultured in W5 solution at 16°C for 18 h in the dark. Then TaALKBH9B-5-GFP protein was purified by GFP-Trap ^®^ M2 magnetic Agarose beads (ChromoTek).

### 2.11 BSMV-induced gene silencing

The optimal VIGS fragment sequence (300 bp) for *TaALKBH9B-5* was obtained using the online VIGS tool [https://solgenomics.net/, (accessed November 1, 2024)]. Insert the fragment into the pBSMVγ vector. The plasmids pBSMVα, pBSMVβ, pBSMVγ, pBSMVγ: *TaALKBH9B-5*, and pBSMVγ: *TaPDS* were linearized using specific restriction enzymes, respectively. Linearized plasmids were transcribed to RNA using the Ribo MAXTM Large Scale RNA Production Systems-T7 and the Ribo m^7^G Cap Analog (Promega). The transcripts were mixed with FES buffer (0.06 M potassium phosphate, 0.1 M glycine, 1% bentonite, 1% sodium pyrophosphate decahydrate, 1% celite, pH 8.5) at 1: 1: 1: 7 (Zhang et al., 2019). Then 10 μL of the mixed transcript was inoculated with the second leaf of the wheat plant at the two-leaf stage by friction (Yang et al., 2020). Wheat plants inoculated with FES buffer were used as a negative control, and plants inoculated with the phytoene desaturase gene exhibiting a typical photobleaching phenotype were used as positive controls. Inoculated wheat seedlings were grown for 24 h under dark conditions of 25°C and 70% relative humidity. After 7 days of growth under 16 h light/8 h dark photoperiod, total RNA was extracted from the hearted leaves and stored at -80°C for detection. The primer sequences used in this study are listed in **Supplementary Table S2**.

### 2.12 RNA dot blot

The nucleotides of the extracted treatment and control groups were sequentially diluted and spotted on the Hybond-N^+^ membrane. UV crosslinking was then performed at UV 254 nm at 0.12 J/cm^2^. After blocking with 5% skim milk for 1 h, the membrane was incubated overnight at 4°C in TBST buffer containing anti-m^6^A antibody (synaptic system). Subsequently, the membrane was washed 3 times with TBST buffer and conjugated with HRP-conjugated secondary antibody (1: 5000, Abbkine Scientific Co., Ltd., California, USA, Cat. No. A21010) for 1 h. Visualized using ImmobilonTM Western HPR substrate Luminol Regeant (Merck Millipore). Further stained the membrane with methylene blue (G1301; Solarbio) to quantify RNA within the membrane (Zhang et al., 2022).

### 2.13 Expression of *TaALKBH9B-5* under hormones and abiotic treatments

For the cold and heat treatments, wheat plants at the three-leaf stage (Yangmai 158) were exposed to 4°C or 42°C for 12 h. To induce drought stress, the seedlings were transferred to a Hoagland liquid culture supplemented with 20% polyethylene glycol (PEG, Sangon Biotech, Shanghai, China) and incubated for 12 h (Wu et al., 2023). For salinity, the plants were grown in Hoagland liquid culture containing 150 mM NaCl (Sangon Biotech, Shanghai, China) for 12 h. For hormone treatments, wheat plants at the three-leaf stage were treated with 100 μmol L^−1^ abscisic acid (ABA) and 100 μmol L^−1^ MeJA as exogenous sprays, as described in Yu et al (Yu et al., 2019). Wheat treated with distilled water was used as a control. Three biological replicates of the samples were collected at five different time points (0 h, 2 h, 4 h, 6 h, and 12 h). All samples were immediately frozen in liquid nitrogen after harvesting and were stored at −80°C until use. The expression of each gene was determined using RT–qPCR. The results were analyzed using GraphPad Prism 9. 0. 0.

### 2.14 Statistical analysis

Statistical analyses were performed using GraphPad Prism 9. 0. 0, and significant differences in the measured parameters were inferred according to Student’s t-test. Significant differences between the two groups of data were evaluated for comparisons (*p* < 0.05).

## 3 Results

### 3.1 Identification of *ALKBH* gene family members in *Triticum aestivum*

To identify members of the wheat *ALKBH* gene family, the BLASTP approach was utilized and employed 14 ALKBH protein sequences from Arabidopsis as a reference. Whole-genome comparisons were conducted for wheat, and the results were further validated through reciprocal BLASTP analysis (**Supplementary Table S1**). Based on these analyses, 30 genes were identified with a similarity greater than 60% between wheat and Arabidopsis. **Table 1** provides a summary of the gene IDs, protein sizes, molecular weights, isoelectric points, and subcellular localization predictions. The sizes of the TaALKBH proteins varied from 255 to 649 aa, the molecular weights varied from 28.82 to 69.07 kDa, and the isoelectric points ranged from 4.97 to 10.33. The protein encoded by *TraesCS4D02G230700* was the longest and presented the highest molecular weight (69.07 kDa), whereas *TraesCS7D02G217000* encoded the shortest protein with the lowest molecular weight (28.82 kDa) (**Table 1**).

**Table 1:** Protein characteristics of the predicted *ALKBH* candidate genes in *Triticum aestivum*.

Gene ID	Size(aa)	MW	PI	Instability Index	SLP
		(KDa)			
TraesCS3A02G028300	367	40.64	6.42	49.54	Chloroplast
TraesCS3B02G018500	365	40.29	6.68	47.76	Chloroplast
TraesCS3D02G017600	370	40.8	6.68	48.07	Cytoplasmic
TraesCS5A02G470000	461	50.06	9.35	52.49	Nuclear
TraesCS5B02G482500	394	42.59	9.38	54.53	Chloroplast
TraesCS5D02G482900	503	54.25	9.25	62.02	Nuclear
TraesCS7A02G215100	257	29.02	9.51	43.77	Nuclear
TraesCS7B02G122300	256	28.97	9.75	37.95	Nuclear
TraesCS7D02G217000	255	28,82	9.69	42.15	Nuclear
TraesCS3A02G097700	284	31.72	6.34	48.99	Extracellular
TraesCS3B02G113400	283	31.42	7.24	42.46	Extracellular
TraesCS3D02G098200	335	37.1	8.45	51.51	Nuclear
TraesCS6A02G334700	268	29.62	4.93	50.03	Cytoplasmic
TraesCS6B02G365100	267	29.4	5.22	45.51	Cytoplasmic
TraesCS6D02G314200	267	29.27	5.11	47.8	MitochondriTaAL
TraesCS2A02G436700	348	37.79	8.32	47.53	Chloroplast
TraesCS2B02G456500	348	37.66	8.32	48.27	Chloroplast
TraesCS2D02G433200	348	37.76	8.52	50.04	Chloroplast
TraesCS2A02G568300	343	37.05	9.38	56.53	Nuclear
TraesCS2B02G628500	346	37.46	9.29	57.86	Nuclear
TraesCS2D02G579400	315	33.39	10.33	59	Nuclear
TraesCS4A02G378300	490	54.43	8.28	55.09	Nuclear
TraesCS7A02G081300	612	67.47	6.32	56.35	Nuclear
TraesCS7D02G075200	391	43.57	9.44	54.47	Nuclear
TraesCS1A02G251400	566	61.4	5.9	53.15	Nuclear
TraesCS1B02G262000	565	61.34	5.97	52.4	Nuclear
TraesCS1D02G250900	559	60.73	6.04	52.84	Nuclear
TraesCS4A02G073400	648	69.07	8.75	63.24	Nuclear
TraesCS4B02G229600	632	67.82	9	60.44	Nuclear
TraesCS4D02G230700	649	69.22	8.78	61.33	Nuclear

aa, amino acid; MW, molecular weight; Da, Dalton; PI, isoelectric point; SLP, Subcellular Localization Prediction.

**Figure 1:**
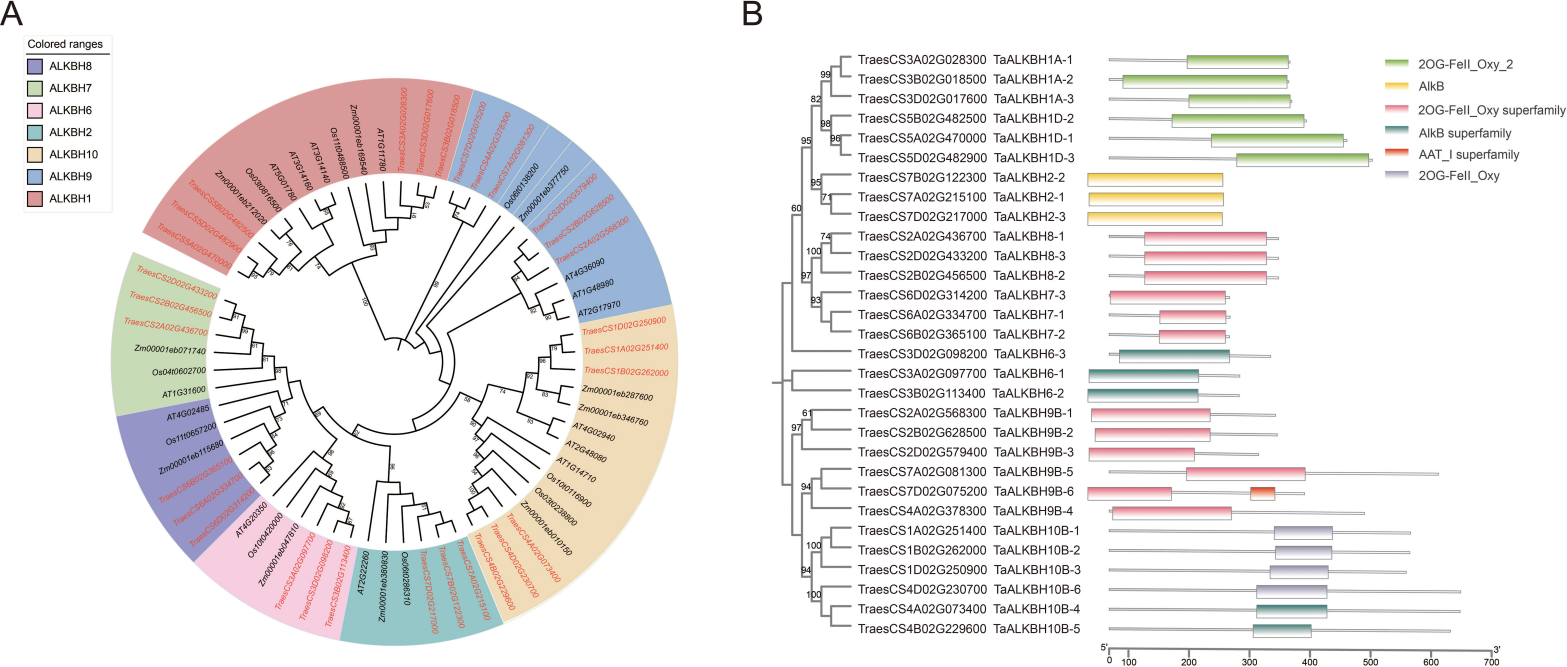
Phylogenetic and conserved-domain analyses of *TaALKBHs*. **(A)** Phylogenetic analysis of ALKBH family proteins in *Arabidopsis thaliana*, *Triticum aestivum*, *Oryza sativa*, and *Zea mays*. An unrooted phylogenetic tree was constructed via the maximum likelihood method implemented in MEGA-X software, with 1000 bootstrap replicates. Distinct colors are utilized to denote different groups. **(B)** Conserved-domain analysis of TaALKBHs. The various domains are depicted using boxes of distinct colors.

A phylogenetic tree was constructed utilizing MEGA-X to examine the phylogenetic relationships of ALKBH proteins across various species. The analysis encompassed 14 protein sequences from Arabidopsis (diploid), 30 from wheat (hexaploid), 9 from rice (diploid), and 11 from maize (diploid) (**Figure 1A**). The TaALKBH, OsALKBH, and ZmALKBH proteins exhibited high homology with the Arabidopsis AtALKBH proteins. Consistent with expectations, the ALKBH proteins from these four species were organized into seven distinct branches: ALKBH1, ALKBH2, ALKBH6, ALKBH7, ALKBH8, ALKBH9, and ALKBH10.

These TaALKBHs were named based on their homologous sequences in Arabidopsis (**Figure 1B**). All TaALKBH proteins exhibited conserved domains, which included 2OG-Fe (II)_Oxy, 2OGFe (II)_Oxy2, the 2OG-Fe (II)_Oxy superfamily, ALKB, and the AlkB superfamily (**Figure 1B**). The homologous proteins within each subfamily displayed identical conserved motifs, implying a potential for functional redundancy.

### 3.2 Analysis of conserved domains and motifs in *TaALKBHs*

**Figure 2:**
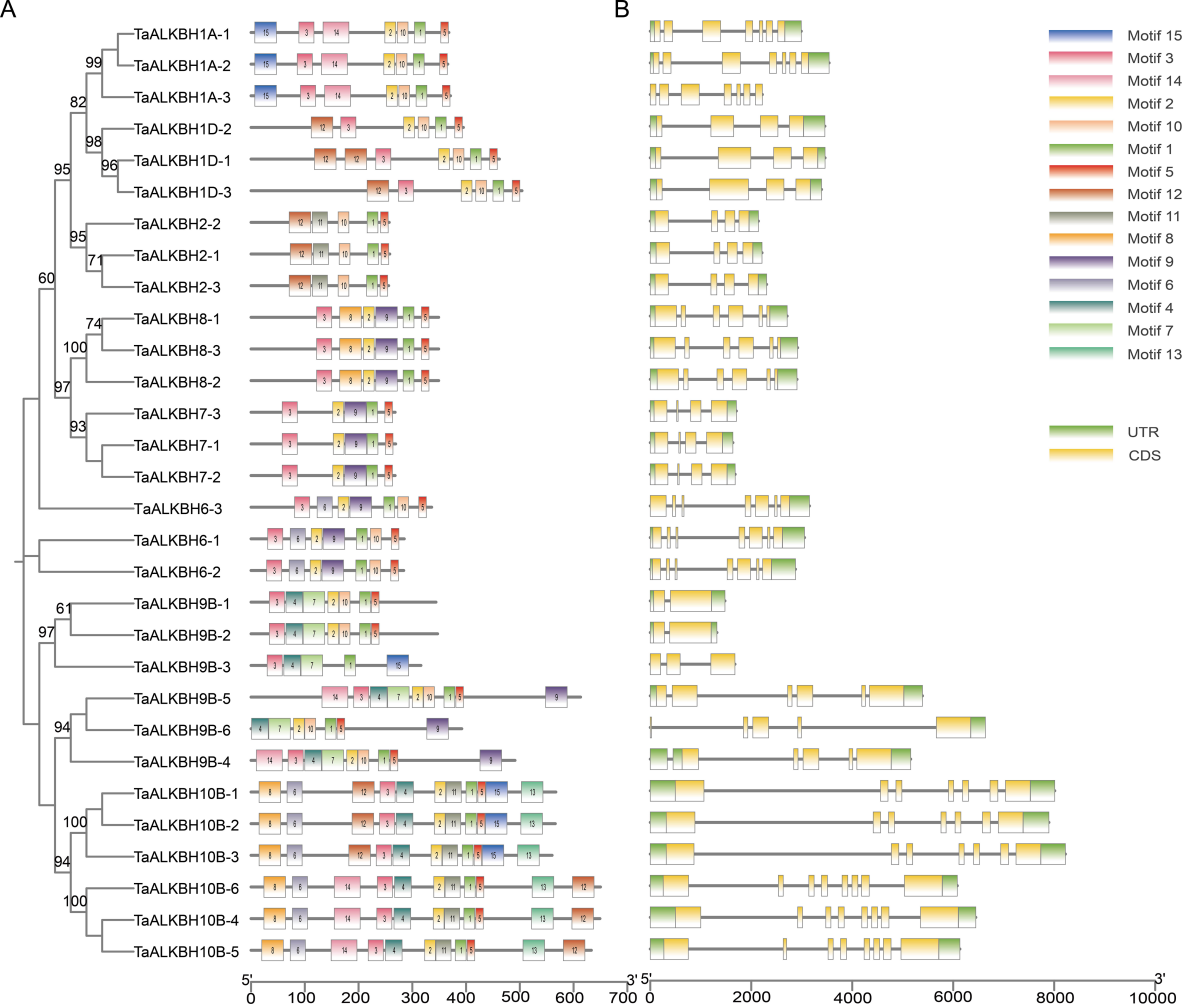
Analysis of conserved motifs and gene structures in TaALKBHs. **(A)** identification of conserved motifs in TaALKBHs. **(B)** distribution of gene structures in TaALKBHs.

Conserved motifs are indicative of gene functions. An analysis of the amino acid sequences of the 30 TaALKBH proteins was conducted to identify conserved motifs using MEME. As illustrated in **Figure 2A**, a total of 15 highly conserved motifs were identified within these proteins. Notably, Motif 1 and Motif 5 were present across all subfamilies, while the ALKBH10 subfamily presented the greatest number of motifs. Additionally, Motif 7 was found to be specific to the ALKBH9B subfamily. The observation that members of the same subfamily displayed similar patterns of conserved motifs suggests functional similarities among these proteins (**Figure 2A**). Due to the similar conserved motifs among the genes within each subfamily, their subcellular localization was further analyzed. The predicted results showed that the same subfamily genes had similar subcellular localization (**Supplementary Table S3**). Furthermore, an analysis of the exon-intron structure offered insights into the evolutionary dynamics of gene families. Examination of the genomic DNA sequences of *TaALKBHs* revealed that genes within the same subfamily generally possess a similar number of exons, although variations in the lengths of exons and introns were noted (**Figure 2B**).

### 3.3 Tertiary structure models of ALKBH protein

**Figure 3:**
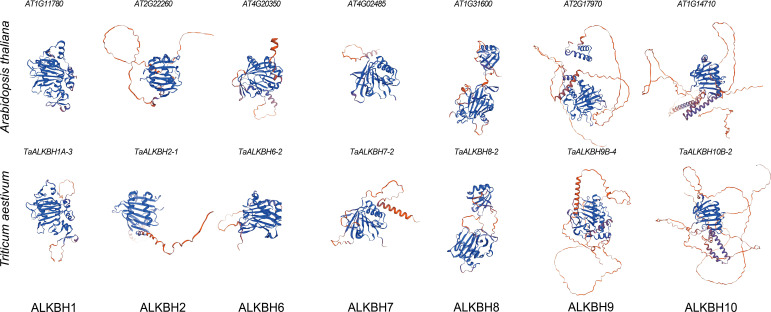
Tertiary structure models of ALKBH protein in Arabidopsis and wheat. Randomly selected a protein from each subfamily and used SWISS-MODEL for structural prediction. Based on QMEAN and GMQE, the model with the optimum results was selected.

The tertiary structure models of proteins are fundamentally associated with their functional capabilities. To construct tertiary structure models of the wheat TaALKBH protein, a representative from each subfamily was randomly selected for modeling purposes. The tertiary structure models of the ALKBH protein from Arabidopsis were utilized as a reference. The results indicated that the tertiary structure models of ALKBH1, ALKBH2, ALKBH6, ALKBH7, ALKBH8, and ALKBH10 from both species displayed significant similarities, whereas distinct differences were noted in the case of ALKBH9 (**Figure 3**). This result revealed the structural diversity of the ALKBH in the two species.

### 3.4 Genomic localization and collinearity analysis of *TaALKBHs*

**Figure 4:**
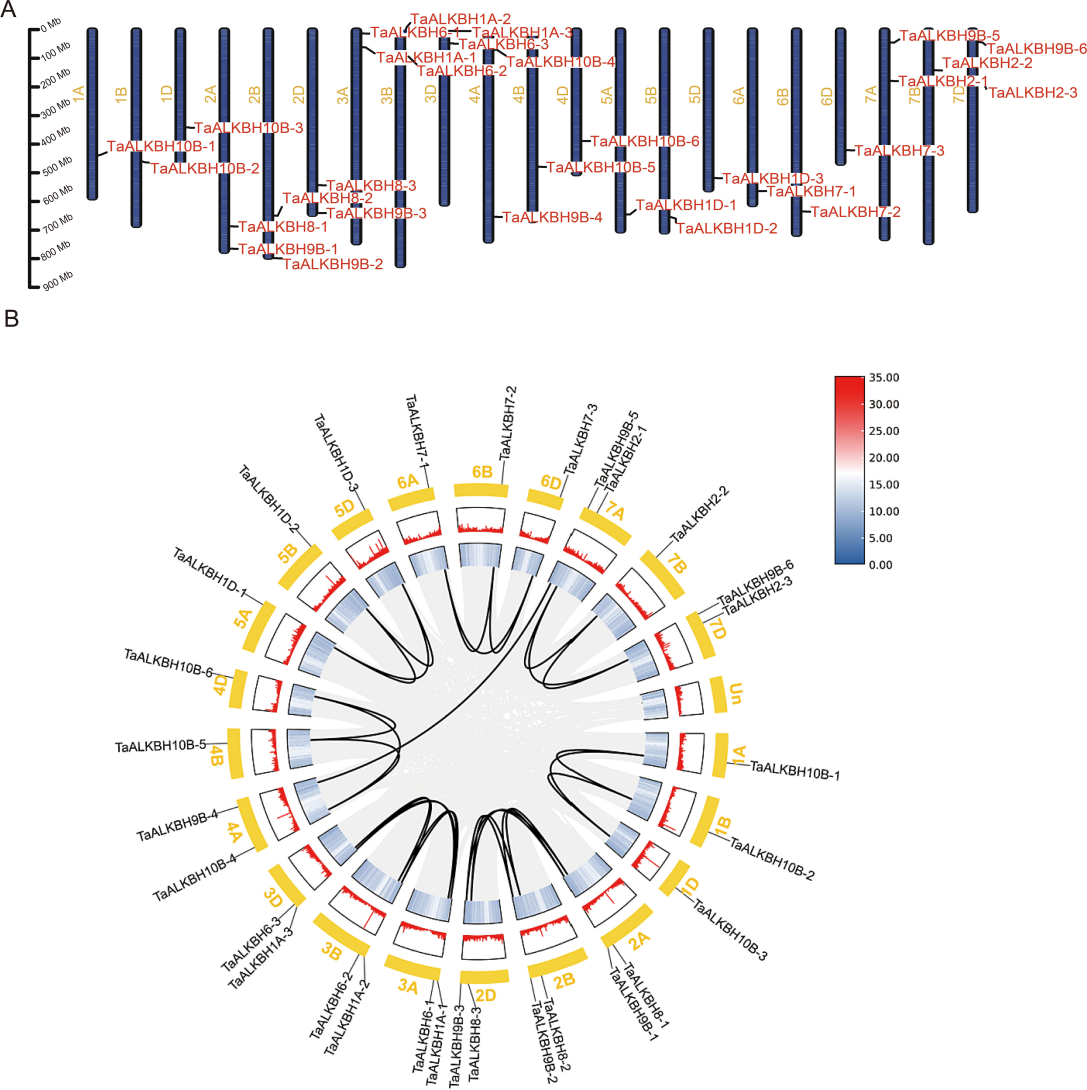
Genomic localization and collinearity analysis of *TaALKBHs.* **(A)** Distribution of *TaALKBHs* on chromosomes. The leftmost scale shows the chromosome length. Chromosomes are represented by green bars. **(B)** Concentric circles, from outer to inner, show (1) *Triticum aestivum* chromosomes, (2) GC content, (3) gene density, and (4) syntenic blocks; the 28 black lines represent collinear pairs of *TaALKBHs*.

The *TaALKBH* genes exhibited a random distribution across the 21 wheat chromosomes, with a majority situated in either the proximal or distal region (**Figure 4A**). Specifically, chromosomes 2A, 2B, 2D, 3A, 3B, 3D, 7A, 7B, and 7D each harbored two *ALKBH* genes, whereas chromosomes 1A, 1B, 1D, 4A, 4B, 4D, 5A, 5B, 5D, 6A, 6B, and 6D each contained a single gene. Regarding the gene density of the whole wheat genome, *TaALKBH1A-1*, *TaALKBH1A-2*, *TaALKBH1A-3*, *TaALKBH9B-1*, *TaALKBH9B-2*, and *TaALKBH9B-3* were located in low-gene-density telomeric regions, which may indicate their role in gene duplication and evolutionary processes (Zhao et al., 2024). Gene duplication events were assessed via BLASTP and MCScanX, and the percentage of wheat genes was incorporated into the collinearity analysis. Among the 30 *TaALKBH* gene members, 28 segmental duplication pairs were identified (**Figure 4B**). In the context of genetics, the *Ka/Ks* ratio serves as an indicator of selective pressure acting on protein-coding genes (Hurst, 2002). Chromosomal covariance and synteny analyses revealed 28 putative paralogous genes (Ta-Ta) in the wheat genome. The *Ka/Ks* ratios for these genes ranged from 0.0505 to 0.5135. All the duplicated *TaALKBH* gene pairs presented *Ka/Ks* values of less than 1, suggesting a lack of strong positive selection. However, one gene pair presented a *Ka/Ks* value exceeding 0.5, indicating weak positive selection. A majority of the gene pairs presented *Ka/Ks* values less than 0.5, implying that the pairs had undergone purifying selection. The divergence time (T) was estimated via the formula T=Ks/(2∗9.1∗10^−9) million years ago (Mya). By this analysis, 28 pairs of homozygous (Ta-Ta) individuals were identified, with T values ranging from 2.342 to 13.241 Mya (**Table 2**).

**Table 2:** Ks, Ka, and *Ka/Ks* values calculated for paralogous *ALKBH* gene pairs (*T. aestivum–T. aestivum*).

Paralogous		Ka	Ks	Ka/Ks	T(Mya)
TaALKBH10B-1	TaALKBH10B-2	0.0074	0.0703	0.1053	3.865
TaALKBH10B-1	TaALKBH10B-3	0.0039	0.0778	0.0505	4.277
TaALKBH9B-1	TaALKBH9B-2	0.0221	0.1153	0.1921	6.334
TaALKBH8-1	TaALKBH8-2	0.0203	0.0949	0.2139	5.214
TaALKBH9B-1	TaALKBH9B-3	0.0249	0.1263	0.1973	6.941
TaALKBH8-1	TaALKBH8-3	0.0170	0.1327	0.1283	7.291
TaALKBH8-2	TaALKBH8-3	0.0135	0.1495	0.0906	8.217
TaALKBH9B-2	TaALKBH9B-3	0.0274	0.0820	0.3339	4.507
TaALKBH6-1	TaALKBH6-2	0.0333	0.1185	0.2807	6.512
TaALKBH1A-1	TaALKBH1A-2	0.0244	0.1440	0.1696	7.909
TaALKBH6-1	TaALKBH6-3	0.0228	0.0871	0.2619	4.785
TaALKBH1A-1	TaALKBH1A-3	0.0132	0.0556	0.2378	3.054
TaALKBH6-2	TaALKBH6-3	0.0156	0.0730	0.2138	4.01
TaALKBH1A-2	TaALKBH1A-3	0.0158	0.1164	0.1356	6.394
TaALKBH10B-4	TaALKBH10B-5	0.0147	0.0620	0.2379	3.405
TaALKBH10B-4	TaALKBH10B-6	0.0111	0.0458	0.2426	2.519
TaALKBH9B-4	TaALKBH9B-5	0.0161	0.0668	0.2404	3.672
TaALKBH10B-5	TaALKBH10B-6	0.0070	0.0619	0.1126	3.402
TaALKBH1D-1	TaALKBH1D-2	0.1064	0.2410	0.4415	13.241
TaALKBH1D-1	TaALKBH1D-2	0.0602	0.1172	0.5135	6.437
TaALKBH1D-2	TaALKBH1D-2	0.0849	0.2215	0.3832	12.173
TaALKBH7-1	TaALKBH7-2	0.0217	0.0708	0.3063	3.888
TaALKBH7-1	TaALKBH7-3	0.0233	0.0944	0.2474	5.184
TaALKBH7-2	TaALKBH7-3	0.0083	0.0426	0.1940	2.342
TaALKBH2-1	TaALKBH2-2	0.0250	0.0893	0.2798	4.904
TaALKBH2-1	TaALKBH2-3	0.0193	0.0550	0.3517	3.02
TaALKBH2-2	TaALKBH2-3	0.0310	0.1111	0.2791	6.105

### 3.5 Functions of cis-acting elements in the promoters of *TaALKBHs*

**Figure 5:**
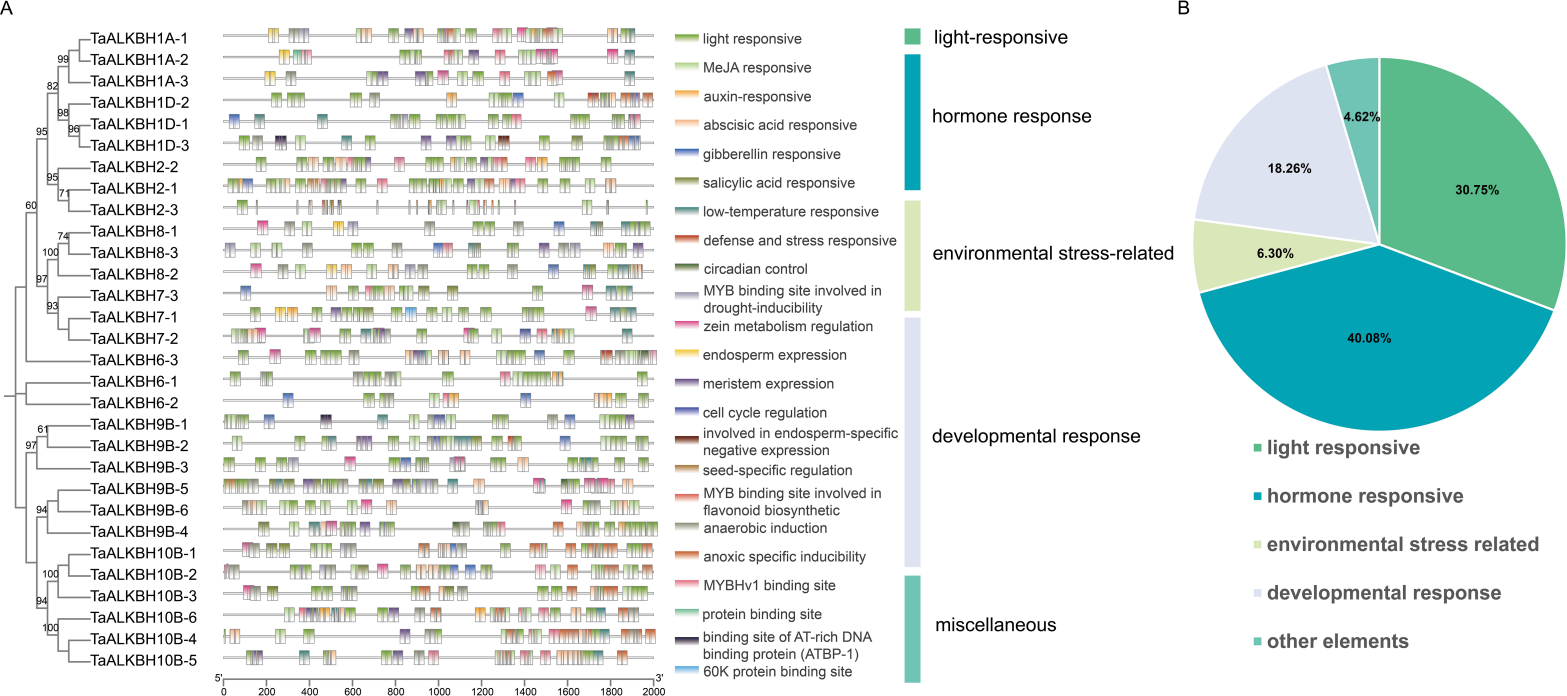
Functions of cis‐acting elements in the promoters of *TaALKBHs*. **(A)** Phylogenetic analysis of *TaALKBHs*. Cis‐acting element distribution in the *TaALKBH* promoters. Different cis-acting elements are represented by different colors. **(B)** The classification of the cis‐elements and the proportions of different types of cis‐elements. The 953 cis-elements were divided into five groups, including 293 light-responsive elements, 382 hormone responsive elements, 60 environmental stress-related elements, 174 developmental responsive elements, and 44 other elements.

To improve the understanding of the potential involvement of *TaALKBHs*, cis-acting elements of 30 *TaALKBHs* were predicted through PlantCARE service using the 2000 bp promoter regions. This analysis revealed 23 functional classes of cis-acting elements associated with *TaALKBHs*. Depending on the different response conditions, these cis-acting elements could be categorized into five groups: light response, hormone response, environmental stress-related, developmental response, and miscellaneous elements (**Figure 5A**). Notably, hormone-response elements were present in all the promoters of the *TaALKBHs*, constituting 40.08% of the total identified cis-acting elements (**Figure 5B**). Among the hormone response elements, those associated with ABA, methyl jasmonate (MeJA), auxin, salicylic acid, and gibberellin presented a relatively high prevalence. The most frequently identified elements related to environmental stress were the low-temperature response elements. Furthermore, cis-acting elements related to developmental responses, as well as defensive and stress responses, were also detected. The significant abundance of light, MeJA, auxin, and gibberellin response elements in the promoters of the *TaALKBHs* suggest that the expression levels of these genes may be modulated by light exposure and various plant hormones, potentially influencing wheat development and response to environmental stimuli.

### 3.6 Tissue-specific differential expression of *TaALKBHs*

**Figure 6:**
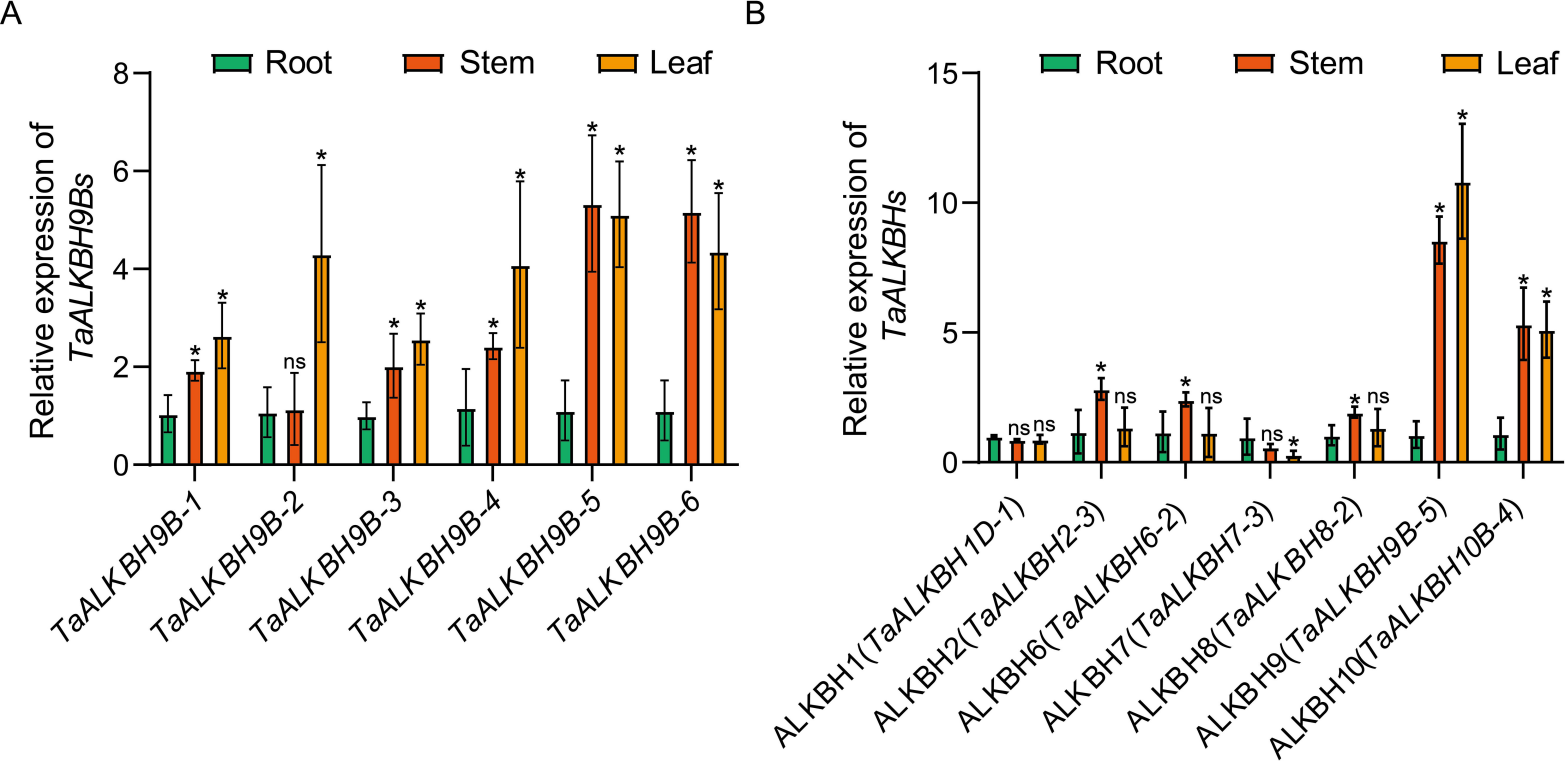
Tissue-specific differential expression of *TaALKBHs.* **(A)** Relative expression levels of *TaALKBH9Bs* genes in roots stems, and leaves. **(B)** Relative expression levels of *TaALKBHs*. The genes were randomly selected from each subfamily. All of the results shown were normalized to *TaCDC* expression as an internal control. Each value represents the mean ± SE of three replicates, and the asterisks represent significant differences between the test group and the control group (* p < 0.05, ns indicating no significant difference, Student’s).

To enhance the understanding of the functional roles of *TaALKBHs*, it is essential to delineate their expression profiles across different tissues. In wheat at the three-leaf stage, the plant is categorized into three distinct parts: roots, stems, and leaves. The physicochemical characteristics of each member of the ALKBH subfamily, as detailed in **Table 1**, correspond with the similarities identified in the conserved domains among the members of this subfamily, as depicted in **Figure 1B**. Additionally, the distribution of gene structure, and the conserved motifs present in each member of the ALKBH subfamily, as illustrated in **Figure 2**, demonstrate significant similarities. The expression patterns of each subfamily are hypothesized to exhibit similarities based on these results. Results from RT-qPCR analysis revealed that all members of the ALKBH9 subfamily, which comprises the largest number of members, exhibited high expression levels in both stems and leaves (**Figure 6A**). Consequently, the expression patterns observed in the members of each subfamily are considered somewhat representative of the entire subfamily. To conduct tissue-specific differential expression analysis, one gene was randomly selected from each group (**Figure 6B**). These results indicated that the expression and distribution patterns of *TaALKBHs* across the seven groups displayed significant variability. Notably, there was no substantial difference in the expression levels of *TaALKBH1D-1* and *TaALKBH7-3* in either rhizomes or leaves. In contrast, *TaALKBH2-3*, *TaALKBH6-2*, and *TaALKBH8-2* exhibited the highest expression levels in the stems. Additionally, the expression levels of *TaALKBH9B-5* and *TaALKBH10B-4* in the roots and stems were significantly higher than those observed in the roots alone, with *TaALKBH9B-5* demonstrating the most pronounced expression. These results imply that the expression patterns of *TaALKBHs* are tissue-specific and may correlate with the various developmental stages of the plant.

### 3.7 Identification of the m^6^A demethylase activity of *TaALKBH9B-5*

**Figure 7:**
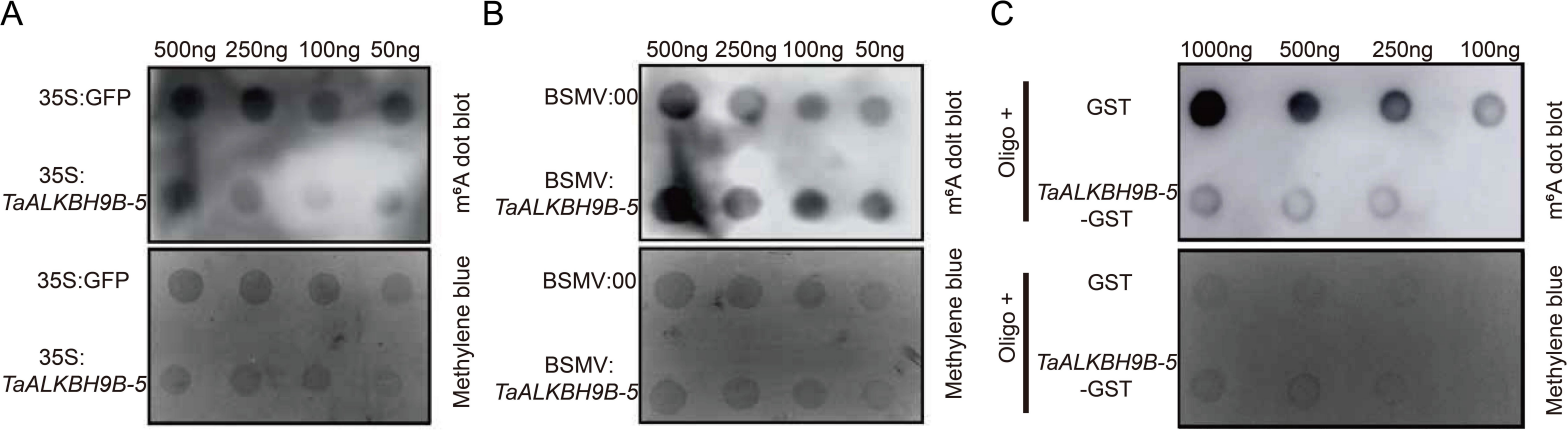
Identification of the m^6^A demethylase activity of TaALKBH9B-5. **(A)** RNA dot blot analysis of m^6^A-modified RNA in the total RNA of 35S: *TaALKBH9B-5*-GFP-transfected wheat protoplasts. 35S: GFP was used to transform the total RNA of the protoplasts as a negative control. **(B)** RNA dot blot analysis of m^6^A-modified RNA in the total RNA after BSMV-mediated silencing of *TaALKBH9B-5*. The total RNA from BSMV: 00 was used as a negative control. **(C)** RNA dot blot analysis of m^6^A-modified RNA *in vitro*. GST and TaALKBH-5-GST were purified from *E. coli*. The GST protein was used as a negative control. The membrane was further stained with methylene blue to quantify the RNA within the membrane.

In Arabidopsis, AtALKBH9B exhibits demethylase activity (Martínez-Pérez et al., 2017). *TaALKBH9B-5* exhibited the most significant variation in tissue-specific expression profiles, it was selected for subsequent validation. Subsequently, the demethylase activity of TaALKBH9Bs was investigated. First, the *TaALKBH9B-5* was inserted into GFP (a vector harboring the green fluorescent protein gene) and overexpressed in wheat cells through protoplast transformation, with GFP overexpression as a control. Total RNA was extracted using alkaline lysis, and m^6^A methylation levels were assessed through m^6^A spot hybridization. As shown in **Figure 7A**, cells overexpressing *TaALKBH9B-5* presented lower levels of m^6^A methylation in total RNA than the control cells (**Figure 7A**). BSMV-mediated gene silencing techniques were employed to silence *TaALKBH9B-5* in wheat. The results indicated that the overall m^6^A methylation level of total RNA in wheat plants with *TaALKBH9B-5* silenced was greater than that in the control group (**Figure 7B**).

Previous studies have reported that ALKBH9B and ALKBH10B exhibit *in vitro* demethylation activities. To obtain the TaALKBH9B-5 protein with *in vitro* activity, the *TaALKBH9B-5* was inserted into pGEX4T-2 (a prokaryotic expression vector encoding GST). *TaALKBH9B-5*-GST was coincubated with the GST vector and Oligo^-^. Compared with that in the GST group, the methylation level in the TaALKBH9B-5-GST supplementation group was lower. These findings suggest that TaALKBH9B-5 functions as an m^6^A demethylase in wheat (**Figure 7C**).

### 3.8 Relative expression level of *TaALKBH9B-5* under abiotic stress

**Figure 8:**
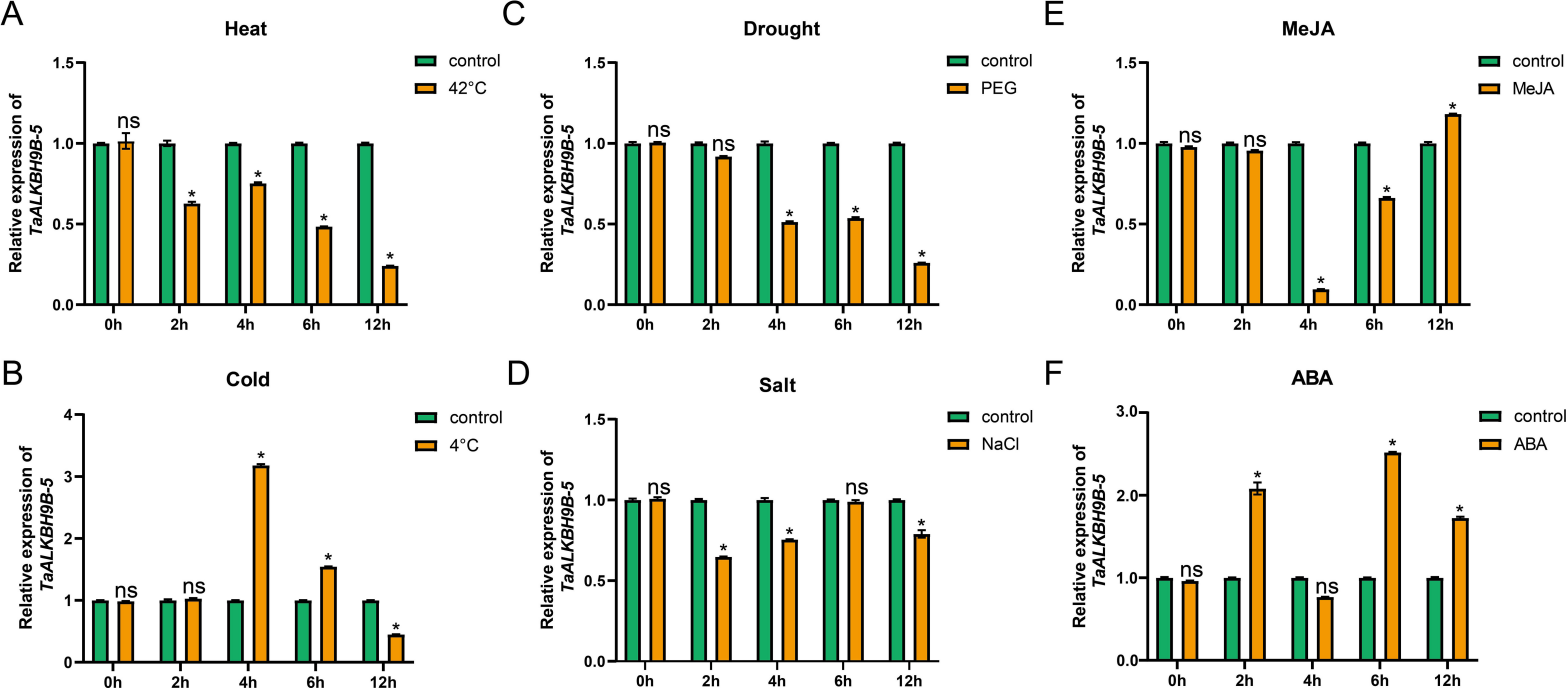
Relative expression level of *TaALKBH9B-5* under abiotic stress. **(A-D)** The relative expression levels of *TaALKBH9B-5* genes in leaves under heat **(A)**, cold **(B)**, polyethylene glycol [PEG, **(C)**], and salt **(D)** stress were quantified using real-time quantitative PCR (RT–qPCR). **(E, F)** RT-qPCR of *TaALKBH9B-5* genes in leaves under MeJA **(E)** and ABA **(F)** treatment. All of the results shown were normalized to *TaCDC* expression as an internal control Each value represents the mean ± SE of three replicates, and the asterisks represent significant differences between the test group and the control group (* p < 0.05, ns indicating no significant difference, Student’s).

The analysis of cis-acting elements indicated that the expression of *TaALKBH9B-5* may be modulated by abiotic stress. To further elucidate the role of *TaALKBH9B-5* in the response of wheat to abiotic stress, the relative expression levels of this gene were assessed in wheat leaves at the three-leaf stage under both normal conditions and various stress conditions, including high temperature, low temperature, drought, and salinity (**Figures 8A–D**). In conclusion, the expression of *TaALKBH9B-5* changed significantly under the four stress treatments. First, the expression of *TaALKBH9B-5* was down-regulated considerably in wheat leaves after 2 hours of treatment at 42°C (**Figure 8A**). Following 4 hours of treatment at 4°C, the expression of *TaALKBH9B-5* was significantly up-regulated in wheat leaves; however, after 12 hours, the expression level of *TaALKBH9B-5* was slightly lower than the control group (**Figures 8B**). Additionally, after 4 hours of treatment with 20% PEG4000, the expression of *TaALKBH9B-5* was significantly down-regulated (**Figure 8C**), whereas *TaALKBH9B-5* was down-regulated considerably after 2 hours of treatment with 150 mM NaCl. These results suggest that *TaALKBH9B-5* may mediate the demethylation of RNA m^6^A and participate in the response of wheat to abiotic stresses.

Recent studies underscore the critical role of plant hormones in the regulation of growth and development. Through gene family analysis, hormone-responsive elements have been identified. To assess hormone responsiveness, three-leaf-stage wheat plants were used for treatments with MeJA and ABA. The cis-acting element analysis revealed the highest number of associated responsive elements (**Supplementary Table S4**). The expression of *TaALKBH9B-5* was measured (**Figures 8E, F**). The findings indicated that *TaALKBH9B-5* exhibited a strong response to both MeJA and ABA; however, the nature of these responses was distinct. Specifically, the expression level of *TaALKBH9B-5* decreased significantly following 4 hours of MeJA treatment (**Figures 8E**), while it increased markedly after 2 hours of ABA treatment (**Figures 8F**).

## 4 Discussion

The ALKBH gene family is integral to plant growth, development, and responses to abiotic stressors through regulating RNA m^6^A demethylation (Tang et al., 2022). The advancement of whole-genome sequencing technologies has significantly improved the accessibility of numerous reference genomes, thereby facilitating the analysis of gene families. *ALKBH* genes have been identified across plant species, including sweet orange, poplar, soybean, and potato (Huang et al., 2024; Zhao et al., 2022; Li et al., 2024). However, the presence of the ALKBH gene family in wheat has not yet been documented. In the present study, a total of 30 *TaALKBH* genes were identified by comparing the wheat reference genome from the Ensembl Plants database with 14 AtALKBH protein sequences from Arabidopsis, employing a BLASTP-based methodology (**Supplementary Table S1**). A phylogenetic tree was constructed that included the 30 TaALKBH protein sequences alongside OsALKBHs, ZmALKBHs, and AtALKBHs (**Figure 1A**), revealing a high degree of homology among the protein amino acids of these species. Furthermore, the TaALKBHs were categorized into seven distinct subfamilies, corresponding to the ALKBH proteins identified in Arabidopsis, specifically ALKBH1, ALKBH2, ALKBH6, ALKBH7, ALKBH8, ALKBH9, and ALKBH10 (**Figure 1A**). The conserved structural domain 2OG-Fe (II)-Oxy suggests these proteins may possess RNA demethylase activity (**Figure 1B**). Members of each subfamily exhibit similar gene structures and conserved motifs, indicating a shared evolutionary origin and functional similarities (**Figure 2**). Most TaALKBHs contain motif 1 and motif 5, which are likely catalytic domains associated with demethylase activity (**Figure 2A**). Significant differences in gene structure among various subfamilies imply potential functional divergence (**Figure 2B**). However, variations in gene structure within certain subfamilies may be attributed to self-mutation events within the wheat genome. These structural alterations could involve changes or loss of gene content in the intron-exon regions, potentially affecting gene functionality. The ALKBH proteins of Arabidopsis and wheat exhibit similar structural characteristics within the same subfamily, whereas proteins from different subfamilies within the same species display considerable structural variation, indicating a diverse structural composition of the ALKBH family across different species (**Figure 3**).

In biological evolution, gene duplication events serve as important evolutionary mechanisms that facilitate alterations in gene function and contribute to gene evolution (Huang et al., 2015). The primary evolutionary patterns observed in land plants include segmental duplication, tandem duplication, and transposition events (Zhang, 2003; Cannon et al., 2004). Notably, evolutionary patterns of identical gene families can vary across species (Qiao et al., 2018; Xu et al., 2020). In the present study, 28 pairs of *TaALKBHs* that underwent gene duplication were examined (**Table 2**). The majority of these pairs resulted from genome-wide duplication events, which contributed to the amplification of *TaALKBHs* (**Figure 4**). To assess the selection pressures acting on *TaALKBHs* and to identify the selection pressures influencing the duplicated gene pairs, Ka, Ks, and *Ka/Ks* ratios were calculated for each homologous gene (**Table 2**) (Hurst, 2002). The *Ka/Ks* values for all duplicated *TaALKBH* gene pairs were less than 1, suggesting the absence of strong positive selection for these gene pairs. These findings underscore the dynamic expansion of *TaALKBHs* and suggest the potential functional diversity or redundancy within wheat. Therefore, the comprehensive identification and analysis of TaALKBHs may provide valuable insights into the functions and mechanisms of RNA m^6^A methylation in plant growth and development.

The analysis of cis-acting elements indicated that all *TaALKBHs* may respond to light, phytohormones, plant growth and development signals, and environmental stresses (**Figure 5**). Among these genes, *TaALKBH9B-5* presented the most significant differential expression between stems and leaves (**Figure 6B**). Previous studies have reported that ALKBH9B possesses m^6^A methyltransferase activity; however, few studies have examined *TaALKBH9B* in wheat. In our investigation, the m6A dot spot analysis results revealed that the RNA m^6^A level in wheat tissues overexpressing *TaALKBH9B-5* was lower than that in the control group (**Figure 7A**). Conversely, the RNA m^6^A level in wheat with *TaALKBH9B*-5 silenced via BSMV-mediated gene silencing was also lower than that in the control (**Figure 7B**), suggesting that *TaALKBH9B-5* can remove m^6^A from mRNA. *In vitro* experiments confirmed this finding (**Figure 7C**). Collectively, these results indicate that *TaALKBH9B-5* functions as an m^6^A demethylase in wheat. In a related study, overexpression of *PagALKBH9B* and *PagALKBH10B* increased the salt tolerance of transgenic lines by mitigating H_2_O_2_ accumulation and oxidative damage through the increased activities of superoxide dismutase (SOD), peroxidase (POD), and catalase (CAT) while also bolstering protection against chlorophyll a/b (Zhao et al., 2022). In Arabidopsis, SG-localized *AtALKBH9B* selectively demethylates the heat-activated retrotransposon element Onsen, facilitating its release from spatial confinement and enabling its mobility (Fan et al., 2023). Silencing of *ALKBH9B* in Arabidopsis has been shown to result in hypersensitivity to ABA treatment during seed germination and early seedling development. The m^6^A residues in the transcripts of ABA INSENSITIVE 1 (ABI1) and BRI1-EMS-SUPPRESSOR 1 (BES1) were removed by *ALKBH9B* following ABA treatment, thereby affecting the stability of these mRNAs (Tang et al., 2022). In this study, cis-acting element analysis revealed that *TaALKBH9B-5* is also responsive to ABA, cold, and drought conditions (**Figure 5**). The relative expression levels of *TaALKBH9B-5* also changed under high-temperature, low-temperature, drought, and salinity conditions, but the expression levels of *TaALKBH9B-5* varied among the different stress treatments (**Figure 8**). The expression of *TaALKBH9B-5* was significantly downregulated after treatment with heat, PEG4000, or NaCl but upregulated after cold treatment (**Figures 8A–D**). Additionally, the expression of *TaALKBH9B-5* was significantly upregulated after treatment with ABA (**Figure 8F**). Drought and salt stress were often associated with ABA, but *TaALKBH9B-5* did not respond consistently to them in these results. This may be because *TaALKBH9B-5* is mainly expressed in leaves, while drought stress and salt stress mainly affect plant roots, and the expression patterns of *TaALKBH9B-5* in different tissues are different and play different roles (Hu et al., 2021). These findings suggest that *TaALKBH9B-5* has considerable potential applications in regulating plant growth, development, and managing abiotic stress responses by mediating the demethylation of RNA m^6^A.

## 5 Conclusions

In this study, an analysis of the *TaALKBH* gene family was performed and identified. TaALKBH9B-5 as an m^6^A demethylase demonstrated a significant upregulation in response to both cold stress and abscisic acid treatment. A total of 30 *ALKBH* genes were identified in the wheat genome. Phylogenetic tree analysis revealed the presence of seven subfamilies. Members within each subfamily exhibited a high degree of amino acid sequence, gene structure, and conserved domain. The *TaALKBH* genes were found to be randomly distributed across 21 chromosomes of wheat. Segmental duplication was identified as the primary mechanism responsible for the expansion of the *TaALKBH* gene family. Predictions regarding the cis-acting elements in the promoters of each gene member indicated that a majority contained multiple hormone response elements and stress response elements. Tissue-specific analysis of the seven subfamilies revealed *TaALKBH9B*-5 exhibiting higher expression levels in stems and leaves than in roots. Dot blot analysis indicated that TaALKBH9B-5 could reduce m^6^A methylation levels under both *in vivo* and *in vitro* conditions, suggesting its potential role as an m^6^A demethylase in wheat. *TaALKBH9B*-5 was significantly upregulated in response to ABA treatment and cold stress. These findings establish a foundation for further investigation into the molecular mechanisms underlying TaALKBH-mediated RNA m^6^A demethylation and its involvement in plant growth, development, and responses to abiotic stresses.

## Data Availability

The original contributions presented in the study are included in the article/**Supplementary Material**. Further inquiries can be directed to the corresponding author.
